# Non-Viral Carriers for Nucleic Acids Delivery: Fundamentals and Current Applications

**DOI:** 10.3390/life13040903

**Published:** 2023-03-29

**Authors:** Sofia Shtykalova, Dmitriy Deviatkin, Svetlana Freund, Anna Egorova, Anton Kiselev

**Affiliations:** 1 Department of Genomic Medicine, D.O. Ott Research Institute of Obstetrics, Gynecology and Reproductology, Mendeleevskaya Line 3, 199034 Saint-Petersburg, Russia; 2 Faculty of Biology, Saint-Petersburg State University, Universitetskaya Embankment 7-9, 199034 Saint-Petersburg, Russia

**Keywords:** non-viral carriers, gene delivery, endocytosis, polyplexes, liposomes, peptide-based carriers, exosomes, microvesicles, nanomaterials

## Abstract

**Simple Summary:**

Gene therapy is one of the most promising approaches to treating various inherited and acquired diseases by delivering genetic constructs into the cells and tissues of the human body. Genetic constructs in general can be described as different DNA and RNA molecules thatcan encode therapeutic proteins or may influence their expression. However, cells and tissues cannot be penetrated easily by DNA and RNA because of their high molecular weight and negative charge. For this purpose, various gene delivery vehicles are studied. The most efficient vehicles are viruses, but they can be dangerous for the organism, so alternate non-viral approaches are intensively studied. This review summarizes recent information on non-viral carriers and the basic requirements for their development.

**Abstract:**

Over the past decades, non-viral DNA and RNA delivery systems have been intensively studied as an alternative to viral vectors. Despite the most significant advantage over viruses, such as the lack of immunogenicity and cytotoxicity, the widespread use of non-viral carriers in clinical practice is still limited due to the insufficient efficacy associated with the difficulties of overcoming extracellular and intracellular barriers. Overcoming barriers by non-viral carriers is facilitated by their chemical structure, surface charge, as well as developed modifications. Currently, there are many different forms of non-viral carriers for various applications. This review aimed to summarize recent developments based on the essential requirements for non-viral carriers for gene therapy.

## 1. Introduction

Gene therapy is considered a promising area of personalized medicine. The successful implementation of gene therapy is due to the deciphering of an effective molecular mechanism to fight pathology, as well as the development of a carrier capable of targeted delivery of therapeutic genetic material to cells. Currently, viral vectors are the most commonly used in clinical trials of gene therapy drugs [1]. However, along with the high delivery efficacy due to the life cycle of viruses, these vectors are immunogenic, and their use can lead to undesirable toxic effects for patients [2,3]. Liposomes, polycations, peptides, and inorganic compounds are examples of non-viral carriers. Non-viral carrier modifications aimed at improving complex stability during systemic delivery, implementing targeted delivery, and increasing transfection efficacy. There are several tricky barriers for complexes with a therapeutic genetic construct, represented by the cell membrane, endosome, and nuclear membrane (in the case of delivery of therapeutic genes). All these barriers, as well as their overcoming by non-viral carriers and their diversity and modifications, will be considered in this review.

## 2. Polyplexes as a Nucleic Acids Delivery System

### 2.1. Nucleic Acids Binding

Since the delivery of “naked” nucleic acids (NAs) shows low efficacy, making this method ineligible for gene therapy patients, the necessity of carrier development is relevant. Most “naked” nucleic acids do not achieve high levels of transfection due to their inability to cross extracellular and intracellular barriers. They are usually eliminated from the bloodstream through systemic delivery and degraded by nucleases in lysosomes or the cytoplasm. Additionally, NAs as anionic molecules usually fail the negatively charged plasma membrane binding and cell internalization, so their charge should be shielded. The ability of any delivery system to bind NAs and form stable particles to prevent degradation and implement efficient delivery is the first critical characteristic.

When forming complexes with non-viral carriers, the type of cargo should be considered. The structure and size of the NA molecule affect binding. Therefore, plasmid DNA is a double-stranded circular molecule with a size ofapproximately 5–10 kb, whereas messenger RNA is linear and single-stranded, with varying sizes and secondary structures [4]. Moreover, because mRNA is much less stable and highly susceptible to RNase cleavage, it should be bound to a carrier immediately [5] or composed of modified nucleotides to increase its stability [6].

One of the most evident and applicable strategies of NA binding is electrostatic interaction. Thus, carriers need to be cationic, as neutral or negative ζ potential results in weak interaction with NAs and the failure of stable structure formation [7]. Polycations that are electrostatically bonded with NA due to interactions between the cationic groups of the polycations and the phosphate groups of nucleic acids are termed “polyplexes” [8]. To achieve the NAs binding, polycations should have a triple positive charge excess or greater [9]. This parameter, however, can vary depending on the chemical properties of the carrier. For instance, to achieve significant transfection efficacy using polyethylenimine (PEI) polyplexes, the charge ratio between the carrier’s nitrogen and the phosphate of NA (N/P ratio) should be five or higher, whereas fluorinated PEI, which can form nanomicelles, can bind DNA at extremely low N/P ratios (one or two) [10]. Thus, the success of NA binding does not directly depend on the value of the cationic carrier charge but to a greater extent on its chemical composition and properties. This observation can also be explained oppositely, namely, that the chemical structure of the carrier determines the charge required for effective binding. Bishop and colleagues demonstrated that the size of polyplexes does not affect their binding ability. Additionally, as the polymer’s binding constant increased, the size of the complexes decreased. This observation is explained by the presence of a larger number of cationic groups that contribute to the denser packing of NAs [11].

Among polycations, a large niche is occupied by positively charged poly- and oligopeptides. Polyplexes containing poly(L-lysine) (PLL) have increased resistance to degradation of both single-stranded and double-stranded DNA, according to Wu and colleagues [12]. The formation of tight polyplexes with PLL depends on the architecture of the polymer (linear or branched) as well as on the environmental conditions [13,14].

Covalent binding, the other mechanism of NA-polyplex formation, has been revisited and rethought over the last few decades. It was considered that nucleic acids could form nanoparticles through covalent bonds with both an inorganic core and a hydrophobic polymer. However, as it was demonstrated, covalent binding was performed indirectly by modification of the 3′-end with biotin, and following avidin binding results in full protection of NA from serum, which is important for systemic delivery [15]. This type of binding is currently known as modification-mediated conjugation [16]. Modifications should be made to the 3′ or 5′-ends of NA to minimize the impact on its functional activity. Furthermore, these modifications should not affect the other functions of the carrier system (e.g., endosomal escape, delivery to the nucleus) or cell components (e.g., intracellular protein complexes).

Another limitation of nucleic acids binding for the delivery into the cell is the ability of the carrier to release the cargo while it has reached its destination. The modifications to carriers that stimuli-responsive cargo release are of great interest. Such modifications can be redox-sensitive disulfide bonds and glutathione moieties, pH- and reactive oxygen species (ROS)-sensitive, or enzyme-triggered systems [17,18,19].

### 2.2. Cellular Membrane Crossing

#### 2.2.1. Cell Membrane Binding

**Figure 1:**
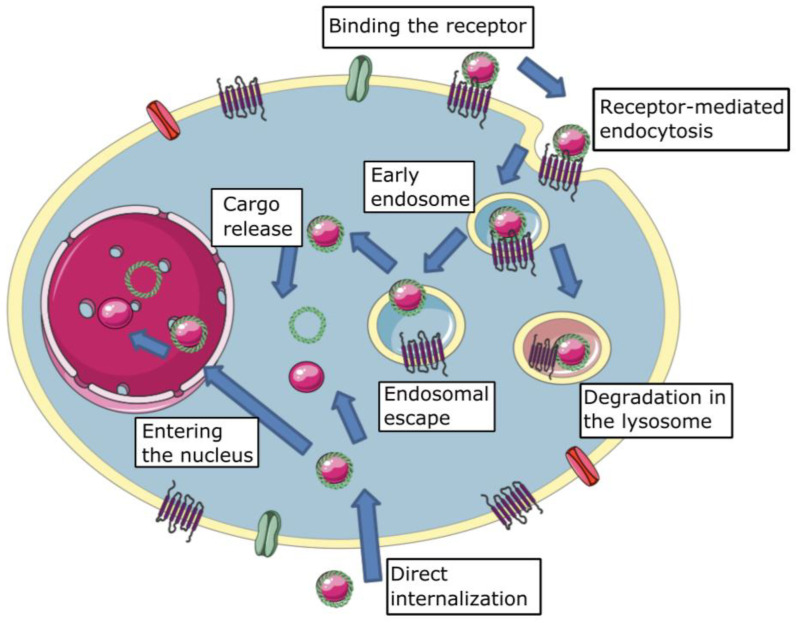
Intracellular barriers to non-viral NA delivery.

One of the major barriers to effective transfection is cellular membrane crossing (Figure 1). The first step in non-viral vector cellular internalization is cell membrane binding. It could be achieved in two ways: (1) specific receptor-ligand interaction and (2) non-specific interaction. Non-specific interaction is most often understood as charge interaction—a negatively charged outer surface of the plasma membrane and positively charged complexes. Complexes are modified by specific ligands to achieve target gene delivery and enhance it in the event the receptor is overexpressed in the tissue. For instance, activated macrophages known to overexpress folate receptor β can be transfected with complexes containing folic acid to treat rheumatoid arthritis [20].

Proteoglycans (PGs) cover the surface of the cell and help bind stable complexes, which leads to successful uptake.There is also evidence that glycocalyx components such as heparin, heparin sulfate, and chondroitin are important for cell binding [21]. Heparan sulfate proteoglycans (HSPGs) are transmembrane proteins that carry several glycosaminoglycans and can promote lipoplex and polyplex uptake, being non-specific receptors. There are two main families of HSPGs: syndecans and glypicans. Glypicans have been identified as Wnt and Hedgehog signaling pathway regulators [22], but not as potential receptors for gene therapy due to potentially deleterious effects. Conversely, syndecans promote the internalization of positively charged particles such as viruses and growth factors. Additionally, cationic complexes are attached to the cell membrane through direct charge interaction. Obviously, glucosamine glycans (GAGs) are not the only anionic molecules that facilitate the internalization of gene-carrying nanoparticles. Even cells without them have sufficient uptake, which means the other molecules on the cell surface, such as glycolipids, are delivering genes into the cell [23]. The binding of certain anionic molecules leads to different endocytic pathways. In particular, PGs can direct cationic complexes into dynamin- and flotillin-dependent pathways [24]. As a result, different receptors are linked to specific endocytic pathways, which result in different intracellular trafficking and necessitate a specific endosomal escape strategy. GAGs-deficient cells show more efficient DNA uptake due to the assumption that GAGs likely lead to lysosomal compartments after internalization, which causes DNA degradation and lower transfection activity [23]. PGs are not the only negatively charged molecules presented on cell membranes. Other anionic components of the cell membrane are glycosphingolipids and sialated proteins, but their role in complex attachment is still unclear [25].

#### 2.2.2. Direct Internalization

Direct internalization means the passage of complexes through the plasma membrane without the participation of any energy-dependent mechanisms (Figure 1). Ming and colleagues demonstrated that the fusion of lipoplexes (particularly complexes with Lipofectamine2000^®^ and DOTAP) and cellular membrane results in additional cell transfection, whereas polyplexes are unable to cross the membrane directly [26]. Other studies [27] have confirmed the ability of direct internalization. Direct membrane translocation is a well-studied method of internalization for cell-penetrating peptides (CPP). However, direct internalization occurs at high concentrations of CPPs and also requires permanent or temporary cell membrane destabilization [28].

#### 2.2.3. Endocytic Internalization

Mammalian endocytic pathways are classified as follows: (1) clathrin-mediated endocytosis (CME); (2) caveolar-type endocytosis; (3) macropinocytosis; (4) phagocytosis; (5) flotillin-associated endocytosis; and (6) non-classical endocytic pathways (clathrin and caveolae-independent endocytosis) [29]. Endocytic pathways are generally classified as clathrin-dependent endocytosis (clathrin-mediated endocytosis) and clathrin-independent endocytosis (others). Each endocytosis mechanism is thought to be a possible method of non-viral gene delivery complex internalization (Figure 1).

The first studies on how non-viral vehicles can cross membranes showed that endocytosis is necessary for significant internalization, but it is not necessary for cell binding. This was shown using electron microscopy [30,31] and endocytosis inhibitors [32]. Endocytosis is now thought to be a major pathway for complex internalization [33].

#### 2.2.4. Clathrin-Dependent Endocytosis

- Clathrin-mediated endocytosis

PEI polyplexes are successfully taken up by clathrin-mediated endocytosis (CME), which does not result in significant transfection activity (probably due to further failure in endosomal escape) [34,35]. In addition, polyplexes are actively internalized via CME if their concentration is low, since low concentration results in smaller polyplex sizes, which CME prefers [36], except for polyplexes containing PEI, whose molecules can be quite large (25 kDa) [37]. Moreover, CME is the main cellular uptake pathway for lipid-modified polyplexes [37].

One of the important features of targeted delivery is the ability to choose a certain internalization pathway using different carrier system modifications. Some cells use one specific endocytic pathway, so it is essential to choose the appropriate nanocarrier for successful gene delivery. Internalization via a specific endocytic pathway may also be preferable for some carriers due to their ability to manage subsequent steps (e.g., endosomal escape).

Clathrin-mediated endocytosis is known as a specific pathway for various ligands, such as transferrin [38], chemokines [39], etc. These molecules mediate internalization through CME. The ligands provide receptor-mediated endocytosis of nanoparticles, although non-specific internalization is preserved.

#### 2.2.5. Clathrin-Independent Endocytosis

- Phagocytosis

For large (more than 1000 nm) polyplexes phagocytosis is supposedly considered a potential pathway of internalization into the cells. This type of internalization is used by a small number of specialized cells (macrophages, neutrophils, etc.) and some non-specialized tissues, such as epithelial [40]. It has been shown experimentally that the phagocytosis-like pathway provides HSPGs internalization using actin cytoskeleton, PAK1, PKC, and tyrosine kinases [41]. It was shown that undifferentiated cells internalize polyplexes using different mechanisms including phagocytosis while mature cells lose this ability [42]. The inhibition of phagocytosis by affecting actin polymerization refutes the hypothesis of lipoplex phagocytosis [35]. Additionally, it should be noted that the disruption of microfilaments decreased the delivery efficacy of PEI polyplexes, even though phagocytosis is not the common mechanism of internalization for this carrier [43].

Internalization of complexes through phagocytosis occurs through the next successive steps: (1) Complexes bind to syndecans via charge interaction; (2) receptors cluster on the plasma membrane in a PKC-independent manner; and (3) PKC mediates the interaction of the actin cytoskeleton with syndecan clusters [44]. Opposite experimental results are showing that some PGs, for instance, syndecan-2, strongly inhibit the uptake of cationic complexes by cells [45]. Some anionic molecules are likely capable of tightly binding the polyplexes and preventing them from attaching to the cell membrane.

There are known receptors that lead to internalization by phagocytosis (for instance dectin-1). However, complexes modified with ligands for these receptors mainly used internalization through another endocytic pathway, such as clathrin-mediated endocytosis [46].

- Macropinocytosis

Macropinocytosis is largely the preferred mechanism of cell internalization bypassing the entry of complexes into the lysosome and nucleic acid enzyme degradation, which significantly increases the transfection efficacy [36,47].

It was shown that the mechanism of internalization of polyplexes largely depends on their concentration. In particular, macropinocytosis is the main pathway at high concentrations of nanoparticles. This may be due to the large size of the resulting polyplexes, which prevent internalization through other mechanisms. It has already been demonstrated [48] that a macropinocytic pathway exists for large (more than 200 nm) Lac-PEI and glycosylated polylysine complexes crossing the cell membrane.Macropinocytosisis a key pathway for the internalization of lipoplexes [49]. It has also been found that macropinocytosis of lipoplexes requires cholesterol. Similar results were obtained by another research group that studied the charge-reversal amphiphile, DNA lipoplex [50]. CPP-conjugated lipoplexes appear to be internalized via micropinocytosis, but only in some cell lines, while in other cells they enter using other mechanisms [51]. PEGylated PLL nanocarriers appear to use lipid raft-mediated macropinocytosis, while unmodified PLL complexes pass through the cell membrane via clathrin-mediated endocytosis [52]. Histidylated polylysine (His-pLK) nanoparticles can be internalized by lipid raft-mediated macropinocytosis due to hydrophobic interactions between histidine clusters and lipid rafts [53]. There is evidence that macropinocytosis is a preferred pathway for the internalization of syndecans-binding cationic lipoplexes. It was shown that the heparin sulfate chains make a large contribution to the internalization of lipoplexes. However, binding to syndecans opens up the possibility of using an additional mechanism of cell membrane crossing [54].

It has been shown that positively charged nanoparticles, which mainly use CME as a cell internalization mechanism, can pass through the plasma membrane by macropinocytosis when both caveolin- and clathrin-dependent pathways are inhibited [55]. In addition, results are showing that inhibition of CME leads to an increase in the efficacy of transfection in some cell lines since macropinocytosis and caveolar-type endocytosis become more actively involved [56].

- Caveolar-type endocytosis

Caveolar-type endocytosis (CTE) is identified as a clathrin-independent endocytic process that includes bulbous-shaped invaginations of the plasma membrane, 50–60 nm in size, called caveolae [57]. This pathway is rather different from CME since CTE predominantly leads not to late endosomes or lysosomes but to caveosomes (early intermediate organelles equivalent to endosomes) [58].

CTE mediates the internalization of polyplexes and ensures high cell transfection efficacy [34], especially for small (less than 100 nm) polyplexes [48].

Chitosan/DNA polyplexes enter the cell mainly through CTE and, to a lesser extent, through macropinocytosis. In the same work, it was found that filipin does not affect chitosan/DNA uptake by caveolae, while genistein largely blocks it. It is known that filipins precipitate cholesterol in the plasma membrane and block caveolae invagination, but for some reason, chitosan/DNA polyplex internalization still takes place [56]. Dendriplexes were shown to enter the cells mainly through CTE (and partially through macropinocytosis), depending on their nanostructure and positive charge density. Additionally, the correlation between the zeta potential of dendriplexes and cellular uptake is non-linear since excess positive charge causes DNA to wrap tightly around dendrimers and interfere with dendrimer/cell membrane interaction [59].

As a natural mediator of HIV internalization, the HIV-Tat peptide is frequently used to improve liposome gene delivery into cells [60]. For liposomes conjugated with the Tat peptide, internalization through caveolar-type endocytosis is shown, while for unmodified ones, CME was used as the main pathway [61]. However, another research group previously identified CME as the main endocytic pathway for Tat-modified liposomes as well as for unmodified liposomes [62]. The difference in the type of endocytosis is possibly related to the size of the nanoparticle, the composition of lipoplexes, and the cell culture.

It should be noted that the pathway leading to the most efficient transfection obviously depends on the chemical structure of polyplexes. Modifications can reduce the dependence of transfection activity on cell membrane cholesterol in the caveolae [37].

#### 2.2.6. Other Types of Endocytosis

Flotillin-associated endocytosis seems to be an additional HSPG-mediated pathway for polyplexes internalization [41]. After binding HSPGs with ligands, the receptors clustered at cholesterol-rich sites and then internalized together with flotillin-1 [63]. Non-classical endocytic pathways include Arf6-dependent, flotillin-1-dependent, Cdc42-dependent, and some others. Internalization of this type occurs upon the receptor interaction and further small membrane invagination, the formation of which does not require a facilitating protein mesh [29].

#### 2.2.7. Difficulties and Promises

Unfortunately, we still have not received detailed and clear mechanisms for cellular membrane crossing by cationic vectors [64]. There are many reasons for it. First, we still do not have specific and effective inhibitors for endocytosis. There is numerous evidence that some inhibitors of endocytosis nonspecifically affect other endocytic pathways in different ways (blocking or activating them) [65,66]. Moreover, endocytosis inhibitors reduce cell viability, which also affects the results. An alternative for such studies is endocytosis-specific gene inhibition, endocytosis-specific markers, and siRNA-based endocytosis-specific gene inhibition [67,68,69].

In gene therapy, it is important to find a specific type of endocytosis for certain non-viral vectors for the following reasons: (1) The cell types often do not exhibit all endocytic pathways, and the use of certain of them can dramatically increase transfection efficacy; (2) some complexes provide sufficient transfection using only one type of endocytosis (e.g., CME), although they can be uptaken by several.

We conclude that the crossing of the cell membrane by non-viral vectors is still an unclear topic, and its study promises great advances in gene therapy.

### 2.3. Endosomal Escape

Entering the endosome is an obligatory step for most complexes consisting of polycations as vectors. In this regard, the success of transfection is limited by the ability of carriers to exit from endosomes before fusion with lysosomes and degradation (Figure 1). The physico-chemical properties of the carriers such as surface charge, size, and buffering capacity play crucial roles in endosomal escape. There are a few strategies to implement the endosomal escape during non-viral gene delivery. While virus vectors use endosome membrane penetration to escape, several non-viral delivery mechanisms have been demonstrated [70]. Hajimolaali and colleagues give the following classification of the main known methods of endosomal escape by non-viral vectors: membrane pore formation, membrane fusion accompanied by cargo release, photochemical disruption, and pH-buffering effects [71,72].

#### 2.3.1. pH-Buffering Effects (Swelling)

One of the most usable modifications is the addition of functional groups that could buffer the pH inside the endosomes, which becomes acidic during maturation. Polymers with pH buffering capacity bind protons, increasing proton influx and subsequent influx of chloride ions and water molecules. The constantly rising osmotic pressure inside the endosome leads to its swelling. This is also known as the “proton sponge effect” [73]. For the first time, this effect was used for PEI-mediated gene delivery because the N-atoms of PEI could be protonated [74].

Chloroquine is one of the well-studied molecules that promote endosomal escape [75,76]. Additionally, it has long been known that the inclusion of this compound in non-viral delivery increases the efficacy of transfection. The mechanism of chloroquine is still not fully understood. There are reports supporting the theory that chloroquine is promoting endosome swelling [75,77]. Other researchers propose that chloroquine has a buffering effect and inhibits the work of lysosomal enzymes [78]. However, the last hypothesis does not explain the absence of rising efficacy of PEI-polyplexes containing chloroquine in THE earliest studies [72,75]. Even though the additional mechanisms of chloroquine’s effects on endosomal escape remain unknown, the fact that swelling and gene delivery are enhanced is undeniable [71].

#### 2.3.2. Membrane Pore Formation

Another strategy for polyplex release into the cytosol is the use of endosomal membrane disruptive molecules. The disruption occurs due to complex molecular changes, including the rearrangement of phospholipid bilayer membrane components [73]. Along with such CPP as pHLIPs [79,80] based on the bacteriorhodopsin protein, this ability was demonstrated for other chemical compounds such as bacterial toxins, in particular, Listeriolysin O [81].

#### 2.3.3. Endosomal Membrane Fusion

Endosome destabilization via the membrane fusion by polyplexes requires the containing of fusogenic peptides. The common mechanism of fusion includes the change of the molecule’s conformation under the influence of the decrease in pH at the endosomal environment, followed by fusion and endosomal membrane destabilization. Hemagglutinin (HA-2), a membrane fusion glycoprotein of influenza virus [82,83], is an example of a fusogenic peptide. This ability was also shown for the HIV-1 fusion peptide gp41 [73,84] and other viral-mimic proteins such as L17E [85] and the amphipathic peptide LK15 [86].

#### 2.3.4. Photochemical Disruption

Currently, research in the field of application and the search for molecules that promote photochemical disruption of endosomal membranes, which began at the turn of the millennium, is ongoing [87,88,89]. The method is based on the use of molecules that have photosensitizer properties. The activation of those molecules by a specific wavelength causes ROS formation, which leads to endosome membrane damage and the release of therapeutic cargo into the cytosol [89]. The application of different molecules as photosensors depends on their absorption properties. Thus, molecules with high absorption, such as aluminium phthalocyanine disulfonate (AlPcS_2a_), are used for in vivo delivery, whereas molecules with low absorption, such as disulfonated tetraphenyl porphine (TPPS_2a_), are used in vitro [90]. However, TPPS_2a_ was photochemically reduced along with other manipulations, yielding the disulfonated tetraphenyl chlorin (TPCS_2a_), which can be used for in vivo applications [90]. This molecule was recently used in clinical trials, where it demonstrated safety and dose-dependent toxicity [91].

It should be noted that any of the described mechanisms of endosomal escape may turn out to be sensitive to different modifications, especially those that aim at surface modifications and increase circulation time [92]. The phenomenon known as “PEG-dilemma” is a prime example of this. The PEG modification of polyplexes and liposomes (resulting in the formation of the structure defined as a polyplex micelle [93]) leads to weak endosomal escape and degradation caused by lysosomal enzymes [94]. This delivery problem nowadays has several solutions, for instance, the incorporation of peptide inserts, which are the targets for matrix metalloproteinases and consequential dePEGylation before entering the endosome [95].

### 2.4. Transport through the Cytoplasm and Nuclear Localization Signals

NAs enter the cytoplasm after being released from polyplexes.They should be bound to the transporter at this point to reach the site of therapeutic action, which could be the nuclei or other organelles (Figure 1). The cytoplasmic dynein seems to be a perfect intracellular transport molecule [96]. Some viruses use dynein during cellular infection due to the special protein sequences with the ability to bind with dynein light chain 8 (LC8) [97]. This feature can be transferred to non-viral carriers to increase their effective delivery of NA in the intracellular compartment. Thus, it has recently been demonstrated that a dynein-binding peptide derived from the Ebola virus (VP35 protein) facilitates efficient cytoplasmic transport and can also be used for BBB passing [96]. PAMAM modified with a dynein LC8 binding module was successfully used during intracellular muscle delivery [98]. Nowadays, this part of the issue of NA transport is gaining popularity among researchers again, and new dynein-binding modifications are being sought [99]. Furthermore, kinesin-binding molecules may also be of interest for intracellular NA transport [100].

While the final destination of siRNA and mRNA is the cytoplasm, the plasmid DNA needs to be transported into the nuclei. DNA containing genes to replenish the lost function needs to be recognized and transcripted as well as maintained in the cell genome to implement a long-term effect. While in stem and tumor cells, the nucleus is disassembled during cell division and the constructs can reach their destination during reassembly, most of the cells in the body are at the terminal stage of differentiation, and their nuclear membrane constitutes a complex barrier. The nuclear membrane, on the other hand, has a number of apertures—nuclear pore complexes—that allow active and passive transport between the cytoplasm and the nucleus. Compounds up to 9 nm in size penetrate the nucleus by passive transport, whereas larger molecules need to be delivered with the expenditure of energy. To be recognized by transporter molecules, this process requires special proteins (importins and GTPases) and a specific label called the nuclear localization signal (NLS) on the delivered DNA. Despite the numerous searches for new nuclei targeting molecules, including proteomics studies, the most commonly used NLS peptide is derived from the simian virus 40 large tumor antigen, contains seven amino acids (PKKKRKV), and mediates the nuclear transport through binding with importin A [101,102,103]. This NLS was recently used as part of chimeric peptide-engineered exosomes, which facilitated nuclear [104]. The proteomic study identified several NLS-containing proteins, including histone H2B, a ubiquitous nucleoside diphosphate kinase NM23-H2, and the homeobox transcription factor Chx10; other sequences are likely to be actively used in the near future [103].

### 2.5. Polyplexes as Systemic Delivery Vehicles

#### 2.5.1. Polyplex Associated Toxicity

Although the main pros and causes of development are the biosafety of non-viral carriers, there are some limitations. Thus, structural features, compound nature, size, and charge can affect the toxicity of the resulting complexes with NA. It should be mentioned that the biodegradation of polymeric gene delivery vehicles is also important because it prevents the accumulation of polyplexes in the tissues, decreasing their toxicity [105,106].

The most widely studied lipid carriers also have some characteristics that can affect their toxic properties. Thus, the chemical structures of the linkers used in lipid carriers make a large contribution to the toxicity of complexes. For instance, ether linkers were shown to decrease biodegradability, which leads to toxicity, while carbamate groups have low toxicity along with the ability to maintain the stability of blood circulation (reviewed in [107]). The polar and hydrophobic domains of cationic lipids also affect toxicity. The single-tailed aliphatic derivates of cationic lipids stipulate more negative effects and negatively affect the transfection efficiency. Cationic lipids, containing cholesterol derivates, can also be toxic due to interactions with protein kinase C and other important enzymes [108].

High molecular weight cationic polymers usually demonstrate greater toxicity. It was shown that poor biodegradability and the lack of an excretion mechanism in high molecular weight (HMW) PEI lead to significant cytotoxicity in vitro [109]. A few years after the occurrence, the hypothesis of necrotic cell death during PEI polyplex internalization was proven [110]. The results of the cytotoxicity mechanism study showed that cell membrane destabilization occurs with some cationic polyplex interactions, such as PEI and PLL, in a dose-dependent manner [111]. Similar HMW toxicity effects were observed for such polymeric carriers as pDMAEMA and PAMAM [112]. The use of low-generation dendrimer structures can help reduce toxic effects [113]. Inorganic nanoparticles used for gene delivery are usually biocompatible; however, their toxic properties can change depending on size and surface coating [107].

One of the primary causes of polyplex negative side effects is a predominance of positive charge. Thus, the toxicity of chitosan is increasing along with the increase in cationic excess [108]. PLL and other cationic polymers are also ineffective due to the charge interactions with serum components, which make them unsuitable for in vivo use [114]. However, to solve this, there are many compounds used to shield the positive charge of polyplexes [115,116,117].

#### 2.5.2. Tumor Targeting

Targeted delivery is one of the most common approaches for effective cancer therapy. The development of the delivery systems modified with targeted ligands and the methods provided for avoiding nonspecific cellular uptake of them are crucial for tumor therapy. Despite the complicated tumor microenvironment, the presence of many types of specific biomarkers in cancer cells allows the use of targeted delivery systems [118]. It should be mentioned that the permeation and retention effect (EPR) is commonly observed in cancers and is thought to be one of the major mechanisms for passive holding of nanoparticles (NPs) 10–200 nm in size, which can contribute to nanocarrier accumulation in cancer tissue [119,120,121]. It is an inefficient and passive strategy since, on average, only 0.7% of the injected nanoparticles can reach the tumor [119]. As a result, using nanoparticles modified with targeted ligands specifically bind to various biomarkers present on target cancer cells is required for successful tumor therapy, as well as other diseases, while reducing ERP side effects. Many different types of tumor-targeted ligands for decorating various nanocarriers have been investigated in recent years. Hyaluronic acid, phenylboronic acid, monoclonal antibodies, various peptides, and small moieties are the most commonly used ligands. Several studies have shown that different delivery systems based on polymers decorated with one of the targeted molecules can effectively and selectively deliver NA and, in some in vivo experiments, inhibit tumor growth and metastasis.

Hyaluronic acid (HA), a natural biomacromolecule, is a linear polysaccharide consisting of alternating units of D-glucuronic acid and *N*-acetyl-D-glucosamine connected by alternating glycosidic bonds –1,4 and –1,3, plays an important role in cell adhesion, growth, and migration [122]. Because HA has a high binding affinity for CD44 receptors, which are overexpressed on many tumor cells, it can be used as a targeting ligand for nanoparticle coating [123]. Kim and colleagues created an HA-CH-NPs/PLXDC1 siRNA delivery system for ovarian cancer anti-angiogenic therapy that targets the CD44 receptor on tumor endothelial cells [124]. The researchers created chitosan particles labeled on the surface with HA through electrostatic interaction. Particles were loaded with siRNA against PLXDC1, which is involved in the promotion of cell migration and invasion of tumor endothelial cells. CD44 and PLXDC1 are overexpressed in endothelial cells and associated with ovarian cancer. The authors demonstrated that these platforms are highly effective and selective, and can protect siRNA from degradation during blood circulation. This study also showed that these carrier systems target siRNA to ovarian cancer-associated endothelial cells, induce effective target gene silencing, and reduce angiogenesis in tumors [124].

It is known that phenylboronic acid (PBA) can react with sialic acid (SA) residues with high affinity to form an annular boronate ester; therefore, SA can be considered a potential target site [125]. Sialic acid receptors are expressed on different components, but many studies have revealed that SA residues are most abundantin malignant carcinoma cells [126]. To avoid interaction between the PBA ligand and SA-associated normal cells in the systemic circulation, Fan and co-workers synthesized a new pH-activated “sheddable” PEG-coated delivery system that was based on the conjugation of PEG-Cat and PBA-terminated PEI (PEI-PBA) through the borate ester formed between PBA and Cat [127]. In this case, PEG was used to “shield” the PBA ligand until the nanocarrier reached the tumor extracellular microenvironment (pH~6.5), after which PBA was exposed and interacted with SA on tumor cells. The nanocarriers have been investigated in vitro and demonstrated improved siRNA uptake, enhanced gene silencing efficacy, and anti-metastatic effects. Furthermore, after intravenous administration, researchers discovered that nanoparticles can accumulate in tumor cells and inhibit tumor growth and metastasis in an orthotopic mammary tumor model [127].

Polyplexes can be decorated with monoclonal antibodies against numerous specific markers highly expressed on the surface of cancer cells. Cai and co-authors designed mPEG-PLGA-PLL-LA/VEGFab nanocarriers containing two targeting ligands, LA and VEGFab, for efficient delivery of microRNA-99a to hepatocellular carcinoma cells [128]. An antibody against anti-vascular endothelial growth factor (VEGFab) can specifically bind to VEGF, while another targeting ligand, LA, binds to the asialoglycoprotein receptor with high affinity. Both are overexpressed in hepatocellular carcinoma (HCC), wherein VEGF is a specific endothelial cell marker and the asialoglycoprotein receptor is a membrane protein on the surface of HCC cells. In vitro studies revealed that the nanocarriers demonstrated outperformed Lipofectamine^TM^ 2000 in terms of delivery, resulting in suppression of proliferation, migration, and invasion of carcinoma cells. Moreover, in vivo experiments showed that the use of nanoparticles in HCC-bearing mice models resulted in the inhibition of tumor xenograft growth with no apparent systemic toxicity observed [128].

Peptides are mostly used to decorate polyplexes to improve the targeted delivery of nucleic acids into various cancer cells. Burks and colleagues have developed polyplexes containing nanoparticles conjugated with Ga-10 (maleimide functionalized gastrin 10 peptide). These polyplexes can selectively target the CK-B (cholecystokinin-B) receptor, which is overexpressed on the surface of cancer cells but not as much on normal pancreatic cells [129]. Treating cancer cells with these delivery systems carrying siRNA against gastrin mRNA leads to the inhibition of the growth and metastasis of pancreatic ductal adenocarcinoma. Taschauer and colleagues utilized a CD49f-binding peptide that was attached to linear PEI via a polyethylene glycol (PEG) spacer [130]. The nanoparticles were loaded with plasmid DNA and can selectively bind to the cell surface marker CD49f, which is overexpressed on various tumor cells. In in vitro experiments, it was shown that plasmid DNA uptake and reporter gene expression were improved in both human and murine tumor cell lines [130]. Furthermore, the polyplexes were used in a syngeneic lung metastasis model in mice via intratracheal aerosolization, which resulted in increased transgene expression in the tumor’s limited area. Durymanov and co-authors have synthesized PEI–PEG polyplex micelles containing MC1SP-peptide, which is a melanocortin receptor-1 specific ligand for targeted nucleic acid delivery to murine melanoma cells [131]. After ganciclovir treatment, the targeted nanoparticles loaded with plasmid DNA carrying the HSVtk gene inhibited tumor growth and prolonged the survival time of the tumor-bearing mouse. Modified polyplexes with small synthetic reTfR (retro-enantio transferrin receptor) ligands have been shown in recent studies to improve gene silencing as well as gene transfer into several receptor-expressing cancer cell lines [132]. A protease-resistant retro-enantio peptide is a short peptide that consists of 12 amino acids in (D)-configuration and in a reversed N to C sequence order [133]. This reTfR peptide was attached to polyplexesvia a monodisperse PEG spacer and used as a targeting module for siRNA and pDNA delivery.

Many small-molecule ligands have shown high binding affinity to the receptors on the surface of various cancer cells. Particularly, folic acid (FA) has also been found to be an optimal ligand for targeting tumor cells due to its low immunogenicity, low toxicity, and high affinity for the folic acid receptor [134]. The FA receptor is overexpressed in most cancer cells, especially in epithelial tumors, so it can be an ideal candidate for targeted delivery of carrier systems [135]. Li and co-authors developed the FA-HP-β-CD-PEI siRNA delivery system based on PEI, which is cross-linked with 2-hydroxypopyl-β-cyclodextrin (HP-β-CD) and folic acid [136]. They used HeLa cells enriched in the FA receptor and siRNA against vascular endothelial growth factor (VEGF). It has been shown that the delivery systems are nontoxic and can effectively bind siRNA and prevent its degradation. In vitro experiments showed that gene silencing efficacy was in the 90% rangeand that expression of VEGF protein was reduced in the presence of 20% serum. In addition, the inhibition of tumor growth and reduced VEGF expression were shown after complex administration via tail vein injection [136]. Biotin can also be used as a targeting ligand for cancer therapy due to its nontoxicity, non-immunogenicity, and ease of modification [137]. Biotin receptors are known to be overexpressed in various cancer cells while being expressed at low levels on the normal cells’ surfaces, thus minimizing the potential for off-target toxicity [138]. Cheng and colleagues created biotin-modified chitosan nanoparticles to stimulate cellular immunity in vivo while inhibiting hepatocellular carcinoma cell proliferation [139]. Application of the biotin-targeted delivery systems led to enhanced gene and protein expression and also prolonged the lifespan of tumor-bearing mice compared to non-targeted nanocarriers. Khan and colleagues decorated the surface of PLGA nanoparticles with *N*-acetylgalactosamine, which has a high affinity for the asialoglycoprotein receptor and can be used to target hepatocytes [140]. In addition, these delivery systems were PEGylated and encapsulated survivin siRNA. The nanoparticle-treated mice showed a 75% improvement in weight loss and a significant reduction in tumor volume compared to untreated mice and mice treated with free siRNA.

#### 2.5.3. CNS Targeting

Nowadays, the molecular mechanisms of many neurological diseases are well studied and offer the opportunity to make gene therapy approaches applicable. However, delivering NA to the central nervous system may be the most difficult challenge of gene therapy implementation. While some viruses are naturally capable of penetrating neurons, non-viral carriers must be modified to overcome the tricky barrier—blood-brain barrier—rather than be stable in a specific immune environment. The blood-brain barrier (BBB) is a tight, three-layer structure consisting of endothelial cells of the blood vessel, astrocyte end-feet, covering the vessel, and pericytes on the basement membrane of the vessel. It is a highly selective structure that prevents substances circulating in the blood from reaching the central nervous system [141]. However, some chemical compounds can both actively and passively pass through the BBB. It was discovered that lipid solubility (with increasing solubility, the passing ability increases) and polar surface area (large surface area and the ability to form more than six hydrogen bonds are limiting factors for BBB crossing) facilitate passive passage through the BBB (reviewed in [142]). The active penetration is carried out by molecular transporters (e.g., P-glycoprotein, MRPs, ABCC1, 2, 4, 5, BRCP, and ABCG2). These molecules are extremely selective and implement the efflux transport of endogenous metabolites [142]. The main role of incoming transporters is given to SLC (solute carrier transporters) molecular family members [143]. They transfer amino acids, glucose, and water-soluble vitamins, and some SLC molecules regulate neurotransmitter homeostasis [144,145].

Due to the great difficulty of CNS carrier development, Ruan and colleagues introduced a summarized delivery cascade named CRITID. It consists of six main steps: blood vessel circulation, BBB recognition, intracellular trafficking through brain endothelial cells, cell targeting after entering the CNS, internalization by target cells, and intracellular drug release [146]. Even though this section of the current review is devoted to binding and crossing the BBB by non-viral vectors, it is worth noting that all steps enumerated in CRITID are important, and their solutions may involve mutually exclusive modifications, as will be demonstrated further.

Following the key features of molecules crossing the BBB passively, it can be considered that non-viral vectors based on liposomes will be preferable as carrier systems. Small (less than 400 Da) hydrophobic polyplexes can be passively internalized through the BBB [141]. However, to improve target delivery, they must also be modified.

Since CNS delivery has been widely studied, several strategies for for overcoming BBB have been proposed. They include BBB disruption, receptor-mediated transport (RMT), adsorptive-mediated transcytosis (AMT), and carrier-mediated transport (CMT), used for nutrition delivery [147].

The BBB can be disrupted in several ways, including the use of active molecules (disruptors), the induction of hyperosmotic shock, and physical influence. Hyperosmotic shock results in the opening of the endothelial tight junctions due to osmotic endotelium shrinkage. For this purpose, a highly concentrated solution of mannitol is usually used [148]. The successful application of combined therapy with mannitol solution was shown [149]. Molecule disruptors that can be used to penetrate the BBB are such biologically active agents as bradykinin and angiotensin [150,151]. Physical approaches to BBB breaching are usually understood as focused ultrasound and photodynamic influence [148]. Despite the encouraging results in clinical practice, breaching the BBB carries risks, so the use of these strategies must be medically justified.

The ability of cationic proteins to bind to the endocytic membrane of brain vessels and internalize through the BBB has demonstrated adsorptive-mediated transcytosis (AMT) [152]. This mechanism results from the charge interaction of polycations with the phospholipid-rich membrane of BBB endothelial cells, which is covered by a glycocalyx of HSPGs, namely glypican and syndecan [153]. As a result, ATM-based strategies for cationic carrier development became actively researched; however, reports of successful results are rare. Cationization, mainly modification by diamines and polyamines, is the most evident way to facilitate delivery through the AMT. For Tat-derived CPP (Tat 47–57, YGRKKRRQRRR) and Syn-B linear peptides, the crossing of the BBB in vivo was shown [154]. The attractive feature of AMT is its ability to internalize large molecules [154]. Nevertheless, represented reports do not erase the crucial disadvantages of using cationic carriers in systemic delivery. The main limitation is the opsonization and interaction of such polyplexes with plasma components, which can lead to immunization and embolization of the vessel. These structures are quickly eliminated from blood circulation, so the delivery efficacy is low. Although other strategies to cross the BBB should be proposed, the observation that cationic peptides and amines can also be transferred to the CNS is valuable.

The most promising strategy for passing non-viral carriers through the BBB is the RMT. This mechanism can be applied using one of two types of chemical modifications: specific ligand sequences or receptor antibody conjugation.

Aside from anti-TfR antibodies, the targeted peptide CRTIGPSVC binds to apo-transferrin (apo-Tf), initializing transcytosis [155]. This peptide affects apo-Tf due to its cyclic iron-mimic form and adopts its iron-bound holo-Tf conformation, which runs the interaction with TfR. AnotherTfR-targeted molecule, the T7 peptide (HAIYPRH), was successfully used to cross the BBB [156]. RVG peptide derivatives, such as rabies virus-derived peptide and RVG29 (YTIWMPENPRPGTPCDIFTN SRGKRASNGGGG), were discovered to specifically bind the BBB nicotinic acetylcholine receptor or γ-aminobutiric acid receptor of BBB [157]. It was shown that Pep-1 peptide (CGEMGWVRC) can cross the BBB and deliver cargo into the brain by binding with interleukin 13 receptor α2 and subsequent endocytosis [158].

The blood-brain barrier is not the only obstruction to CNS delivery. Non-viral carriers must also target a variety of cells beyond the BBB. Several ligands have been proposed for this purpose.Thus, for gene delivery into glioblastoma stem cells, the CD133-targeted RNA aptamer A15 was used [159,160]. Integrin binding was proposed for neuroblastoma cells. The peptide ME27 (RVRRGACRGDCLG) was shown to be able to initialize the αvβ3- and αvβ5-targeted delivery into neuroblastoma in vivo [161]. Tet1 (HLNILSTLWKYR) peptide is highly used for sphingomyelin and ganglioside GT1B and neuron targeting [156,162]. Additionally, the neuron-targeted capacity was shown for peptide Y (GACYGLPHKFCG) [163].

#### 2.5.4. Other Targets

Since gene therapy has various applications (cancer, monogenic diseases, infections, etc.), the need for specific delivery systems is growing. The characteristics and features of targeted cells should allow directed polyplexes internalization. The primary targeting strategy is ligand-receptor interaction followed by endocytosis. All types of vectors can carry the modification of guide molecules, which significantly enhances their safety and the efficacy of gene therapy. Along with tumor and CNS targeting, the search for ligands for specific delivery to other tissues is being actively pursued.

- Muscle targeting

The efficient delivery of polyplexes to the muscle tissue is a task that has become especially relevant in recent years. Skeletal muscles are considered to be an ideal tissue that can be used to produce certain proteins for therapeutic purposes or immunization. However, to obtain the expression of exogenous genes, the injection of polyplexes is not enough. Muscle tissue is one of the most difficult cell types to transfect due to its anatomic structure and lack of specific ligands that promote internalization. Skeletal muscles are represented by bundles of syncytial cells, named myocytes or muscle fibers. Each of them is surrounded by connective tissue cells, endomysium, while the bundles are in turn coated with perimysium [164]. These connective tissue structures significantly obstruct the entry of therapeutic polyplexes into muscle cells. Nevertheless, individual muscle fibers are supposed to persist for most of their lives through their terminal differentiation, providing a stable “factory” for continuous transgene expression [165]. It has recently been demonstrated that muscle cells can produce enough coagulation factor FVII-hFc immunoconjugate to immunotherapeutically suppress tumor growth [166]. Another study investigated the delivery of plasmid DNA to skeletal muscle to produce the single-strand insulin analog for the treatment of insulin-dependent diabetes [167]. In both of these works, low-voltage electropulse was used to achieve high delivery efficacy. As a result, the search for a specific ligand is still necessary. The most well-studied ligands for muscle delivery are the ASSLNIA peptide [168,169] and the A2G80 peptide (VQLRNGFPYFSY), targeting α-dystroglycan (α-DG), expressed on the muscle cell membrane [170,171].

Carrier systems for gene therapy of congenital myopathies and muscular dystrophies are also being developed. This is in addition to the use of muscle delivery for indirect therapeutic purposes. It is worth noting that the treatment of hereditary muscular diseases is complicated by the total volume of muscle tissue, which is half of the total body mass, poor accessibility of deep muscles, and progressive tissue remodeling that is characteristic of the natural course of diseases [172].

In recent years, several works have reported the efficient non-viral carrier system for muscle delivery. Liposomes modified with an A2G80 targeting peptide successfully penetrated muscle cells in vivo [173]. Thus, there is a report of successful plasmid DNA delivery in vivo using polycondenced peptides as non-viral vectors [174].

- Stem cells targeting

Stem cell technology, along with gene therapy, is a powerful tool of personalized medicine. Using stem cells is promising for regenerative medicine due to their multipotent differentiation capacity and immunocompatibility [175]. Internalizing exogenous genes of specialized protein cell markers can influence differentiation processes. To carry out these manipulations properly, a highly efficient carrier system is required. Gonzalez-Fernandez and colleagues compared the three different classes of non-viral carriers for gene delivery—PEI, nanohydroxyapatite (nHA), and the RALA (containing arginine/alanine/leucine/alanine repeating units) amphipathic peptide—to transfect bone marrow-derived mesenchymal stem cells. The most accurate results for inducing chondrogenesis were shown for RALA- and hHA-mediated delivery, while PEI-containing polyplexes failed to induce target phenotype formation [175]. It was shown that gold nanoparticles (AuNPs) conjugated with an antimicrobial peptide (PEP) are an effective delivery system for providing DNA transport to the rat mesenchymal stem cells [176]. Other conjugate materials, such as superparamagnetic iron oxide nanoparticles covered with PLL, were introduced as non-viral carriers for neural stem cells [177]. In another study, ectodermal mesenchymal stem cells were successfully transfected with carbon dot polyplexes derived from porphyra polysaccharide [178]. Induced pluripotent stem cells (iPSC) are specific types of stem cells that have been actively studied in the last decade. The concept of delivering differentiation factors genes into cells holds great promise when compared to the traditional approach of using cultivation medium supplements [179]. Several non-viral carriers, such as mesoporous silica nanoparticles [180], elastin-like polypeptides [181], octadecylamine-based cationic lipid nanoparticles [182], minicircle plasmids [183], etc were investigated for this purpose and further re-differentiation.

- Fibroblasts targeting

Fibroblasts are the major cellular components of connective tissues and another difficult cell type to transfect. However, the availability of these cells due to their ease of isolation, good culturability in vitro and ex vivo, and rapid growth makes them one of the main model objects in cytological and genetic studies [184]. Fibroblasts are also used as the material to obtain the iPSC [185,186,187]. Despite all of the above, fibroblast primary cells are difficult to transfect, which contributes to the active search for an effective carrier [184]. There has been success using the linear-branched hybrid poly(β-amino ester) as a non-viral carrier for fibroblast gene delivery. The reported efficacy is about 93%, which is much higher than for commercial carriers [188]. Chang and co-workers demonstrated successful reprogramming of fibroblasts to induced cardiomyocytes via delivery of reprogramming factors genes by AuNP modified with nonspecific cationic peptide ligand RRRGYC. Cell internalization is mediated by the efficient formation of hydrogen bonds between the guanidine moieties of the ligand and negative residues on the cell surface [189]. The observation that cationic segments highly contribute to the efficacy of gene delivery into fibroblast cells is confirmed regularly. Thus, the high transfection level of primary dermal fibroblast cells was obtained using polycation with a tenfold predominance of a positive charge over negatively charged DNA [190]. The recent works lead to the suggestion that the difficulty of fibroblast transfection is due to the sensitivity of cells to polyplexes’ charges. Polyplexes modified with coating modules, whicheffectively conceal the positive charge, were less effective compared to cationic ones. Another strategy that could be suggested to overcome the first cellular barrier is the use of ligands. Since the efficacy of a non-specific ligand has been shown, this suggests that modifications with only the cationic residues contribute to the efficient uptake of polyplexes by fibroblasts.

## 3. Non-Viral Vectors for Nucleic Acids Delivery

### 3.1. Lipid-Based Carriers

In recent decades, lipid nanoparticles have been widely studied as delivery vehicles for siRNAs, therapeutic genes, and drugs. Recent studies show that lipid-based carriers have significant potential as successful delivery candidates to improve the efficacy of cancer therapy and can also be used as mRNA vaccines for the treatment of some diseases, for instance, COVID-19.

Liposomes, which consist of one or more concentric, closed phospholipid bilayers [191], are a type of lipid-based formulation that is often used in gene therapy. Phospholipids have hydrophobic heads and hydrophilic tails, forming an internal hydrophilic core into which various water-soluble substances can be delivered to cells [192]. In addition, it can also be used to deliver hydrophobic therapeutic agents that are absorbed onto the surface of the liposome or incorporated into a lipid bilayer matrix [193,194]. There are several classes of liposomes such as multilamellar vesicles and unilamellar vesicles, which can be further classified as giant unilamellar vesicles and as small unilamellar vesicles [195,196,197].

Liposomes have two important advantages, such as good biocompatibility and biodegradability, which are due to lipid characteristics [198]. Smistad and co-workers have been studying the toxicity of liposomal formulations using the human buccal cell line TR146 [199]. They reported that cationic liposomes are more toxic in vitro compared to neutral and negative-charge liposomes. The optimal size of liposomes required for efficient delivery can vary from 70 nm to 300 nm [200,201].

Traditional liposomes have serious limitations for biomedical applications because they can aggregate and are rapidly destroyed by the reticuloendothelial system (RES) via opsonization followed by phagocytosis [202,203]. Coating liposomes with PEG has been proposed as a widely used approach to increase the circulation half-life and form a “shielding effect” to exit from the RES (“stealth” liposomes). PEG is an inert, biocompatible, hydrophilic polymer capable of forming a protective layer on the surface of the liposomes and providing repulsive interactions between blood components and the surface of the delivery system [204].

The liposomes are often used to develop cancer drug delivery systems and can be applied for passive and active targeting of tumor tissues. The passive strategy is explained by the “enhanced permeability and retention effect” phenomenon [205]. The tumor tissue is characterized by excessive angiogenesis and large vascular pores in comparison with normal vessels. That allows liposomes to accumulate in the tumor tissue by convection or passive diffusion. The poor lymphatic drainage in the tumor interstitium leads to the retention of nanocarriers. However, the effectiveness of the passive targeting strategy is dependent on several factors, including nanocarrier size and circulation half-life, the degree of tumor vascularization and angiogenesis, the pore size of tumor vessels, which varies depending on the type of tumor, and increased interstitial fluid pressure, all of which are serious limitations for effective cancer therapy [206,207].

The delivery of drugs by active targeting is preferable and does not eliminate the effect of passive targeting. There are two groups of developed liposome delivery systems that use an active targeting strategy. The first group of methods is based on the attachment of certain ligands to the surface of liposomes, for instance, antibodies (immunoliposomes) [208], peptide ligands (peptide-targeted liposomes) [209], aptamers [210], folate [211], and transferrin [212]. The second strategy may involve the use of stimulus-responsive liposomes, which are “smart” liposomal systems that exhibit rapid release of their drug load upon physicochemical or biochemical stimuli. The most common irritants are temperature (thermosensitive liposomes) [213,214], pH (pH-sensitive liposomes) [215], redox potential (redox-sensitive liposomes) [216], light (light-sensitive liposomes) [217], magnetic fields [218], and X-ray radiation [219]. Cardoso and colleagues have demonstrated solid magnetoliposomes responsive to multiple stimuli such as thermal, magnetic, and pH for controlled release of doxorubicin in pathological areas [220]. The development of such liposomes has great potential as prospective drug carriers for cancer therapy.

To study the ability of liposomes to bind and deliver nucleic acids, positively charged lipids were incorporated into the liposome membrane. The cationic liposomes have been considered a large group of liposomes that are potential non-viral delivery nanoplatforms. They usually consist of a cationic lipid derivative and a neutral phospholipid (DOPE). The latter is required by certain cationic lipids to form stable liposomes. Some of the widely used cationic liposome formulations are lipofectin (DOTMA/DOPE 1:1, mol/mol) [221,222]; RPR-120535 [223]; lipofectamine (DOSPA:DOPE, 3:1) [224]; transfectace (DDAB:DOPE, 1:3); transfectam (DOGS) [225], and DC-Chol-DOPE [226]. Lipoplexes are formed when ionic liposomes interact passively with nucleic acids [227]. Lipoplexes can enter the cell by fusion with the cytoplasmic membrane and facilitate the release of NA from endosomal membranes after the absorption of lipoplexes by the cell [227]. However, using these lipidic nanoplatforms has some drawbacks, including toxicity, immunogenicity, and a short half-life in the circulatory system [199,228]. To overcome the shortcomings of lipoplexes, researchers have designed and synthesized various advanced cationic lipid-based nanocarriers: lipid-polycation-DNA complexes (LPD), liposomes-protamine-hyaluronic acid nanoparticles, and stable nucleic acid-lipid particles (SNALPs). They have demonstrated controlled morphology and particle size and provided a good solution for in vitro and in vivo siRNA delivery.

Gao and Huang represented self-assembling LPD nanoparticles for the delivery of DNA plasmids [229]. PEG coating was used to improve these formulations for in vitro and in vivo siRNA delivery [230]. They used calf thymus DNA to encapsulate siRNA in the condensed core. Then the condensed core of cationic polypeptide protamine was coated with DOTAP/cholesterol cationic liposomes. DSPE-PEG, with or without anisamide (AA), was inserted into the lipid membrane, forming a neutral charge and preventing the aggregation. The researchers developed complexes for targeted delivery of AS-ODN (antisense oligodeoxynucleotide) or siRNA against human survivin in lung cancer cells. They demonstrated that formulations with a DSPE-PEG-AA coating can effectively deliver therapeutic cargo into the H1299 cancer cell line, inhibit tumor cell growth, and sensitize them to anticancer drugs [231]. However, the utilization of calf thymus DNA as a part of this delivery system can lead to toxicity and stimulation of the immune system [232]. Researchers from the same laboratory replaced calf thymus DNA with hyaluronic acid, and the resulting complexes were used to deliver siRNA into the tumor [232]. They have demonstrated that HA can also effectively condense siRNA in the presence of protamine but has much lower immunotoxicity compared to calf thymus DNA in LPD nanoparticles. SNALPs are characterized by high cargo encapsulation efficacy and can be designed to promote targeted delivery to specific receptors. Typically, SNALPs have an average size of 100–150 nm, are neutrally charged, and consist of three important components: an ionizable cationic lipid, a non-ionic helper lipid, and a PEG-derivatized lipid [233]. SNALPs are nanocarriers with optimal physico-chemical features for in vivo delivery of therapeutic nucleic acids, such as siRNAs [233].

Several successful studies have been conducted on the use of LNP-based delivery systems as one of the most commonly used non-viral nanocarriers for RNA delivery to targeted cells. LNPs coated with PEG for intratumoral delivery in mice resulted in apoptosis in hepatocellular carcinoma cells without systemic cytotoxicity, according to Jain and colleagues [234]. Lai and co-authors created IL-12-LNP nanoparticles loaded with mRNA of interleukin-12 (IL-12) for the in situ delivery of this cargo in a primary transgenic mouse model of refractory MYC-driven hepatocellular carcinoma [235]. They have shown that the use of the developed complexes led to the suppression of the progression of MYC oncogene-driven hepatocellular carcinoma, which is well distributed within this cancer and has no significant cytotoxicity [235]. Endo-Takahashi and co-authors have created PEG-modified bubble liposomes that trap ultrasonic contrast gas. They have demonstrated that this combination is an effective nanoplatform for pDNA and siRNA delivery in vitro and in vivo [236].

It is known that liposomes can be used not only to deliver NA or drugs but also for the delivery of mRNA vaccines. Persano and colleagues designed a lipopolyplex platform, which consists of mRNA molecules packaged in a polymeric polyplex encapsulated in a phospholipid bilayer shell structure [237]. The developed vaccine was used to deliver mRNA-expressed ovalbumin and tyrosinase-related protein 2 antigens to dendritic cells to stimulate an immune response through Toll-like receptor 7/8 signaling. It has been shown that dendritic cells treated with this vaccine strongly expressed interferon-β and interleukin-12 and demonstrated a high level of antigen presentation capability. They also showed that the use of the created vaccine in murine models with metastatic B16-OVA lung tumors expressing the ovalbumin antigen resulted in a decrease in tumor nodules by 90% and could be a potential immunotherapeutic agent in the treatment of many types of diseases [237].

The lipid-based nanoparticles can also be used for the development of SARS-CoV-2 (severe acute respiratory syndrome coronavirus 2) mRNA vaccines. The mRNA-1273 vaccine by Moderna [238], CvnCoV [239], and CV2CoV [240] based on CureVac’s RNActive platform and BNT162b2/Comirnaty by BioNTech/Pfizer [241] are all known. The LNP component of these vaccines consists of ionizable cationic lipids, neutral lipids, cholesterol, and PEGylated lipids at different molar ratios. In recent research, Li and colleagues have presented a COVID-19 mRNA vaccine based on AA3-DLin LNPs developed by the one-step CALB (Candida antarctica Lipase B) enzyme-catalyzed synthesis method [242]. The authors have reported that utilizing this methodology avoids the drawbacks resulting from the multiple-step chemical reactions applied by Moderna and BioNTech/Pfizer. They have shown that the LNP vaccines exhibit not only a high mRNA delivery efficacy but also a great long-term storage capability at −20°C. The researchers have also stated that this method has a huge potential for developing mRNA vaccines to treat various diseases [242].

Ryu and colleagues used Cas9-RNP enclosed in nanoliposomes conjugated with microbubbles. Such a delivery system transfects dermal papilla cells due to microbubble cavitation under the action of ultrasound, which provides highly efficient and site-specific delivery [243]. Lecithin liposome/Cas9-RNP lipoplexes targeting hepatic cells have been used to treat diabetes type 2. The liposomes contained cholesterol and DOGS-NTA-Ni to facilitate encapsulation of the Cas9-NLS His-tagged RNP as well. PEI was added to Cas9-RNP to compensate for its excess negative charge and improve the formation of lipoplexes [244].

The development of delivery systems for therapeutic agents based on lipid nanoparticles is an ever-growing area of research. Currently, lipid-based nanocarriers are one of the most attractive and commonly used non-viral delivery systems for cancer therapy. The ability to modify them and manipulate their different characteristics makes these carriers potentially versatile for delivering a wide range of compounds. It should be noted that lipid nanoparticles are the most extensively researched class of mRNA delivery carriers [6]. Current mRNA delivery systems are derived from lipid carriers, in particular liposomal forms for small molecule therapeutics [245].

### 3.2. Peptide Carriers

The peptide-based carriers are commonly used in gene therapy studies as potential delivery vehicles. These promising therapeutic tools are relatively stable and have several advantages, including low cytotoxicity and immunogenicity profiles, excellent biocompatibility and biodegradability, and ease of production and modification [246,247]. Many peptides can be used not only as self-sufficient carriers but as functional elements in non-viral delivery systems to increase transfection efficacy and facilitate delivery to target sites. The peptide vectors can be divided into the following types according to their application to overcome biological barriers: CPPs, target peptides, NLS-carrying peptides, and membrane-active peptides.

There are some strategies to formulate peptide-nucleic acid complexes. The interaction between the molecular cargo and peptide can be either covalent or non-covalent. Both approaches have their advantages and disadvantages, and the choice of one type of binding or another usually depends directly on the structure of the interacting molecules. The simplest method is non-covalent interaction since a simple mixing of the two components is required. Peptides are typically designed with positive charges that can bind with negatively charged nucleic acids, neutralizing the charge and inducing the hydrophobic collapse of nucleic acids into condensed nanoparticles [248]. For instance, electrostatic interactions between CPP and nucleic acids, as well as the complex formation of amphiphilic peptides MPG and Pep-1 with a therapeutic agent [249]. The most common method is covalent bonding, and both direct and indirect isolation via various transport systems, such as polymeric carriers or liposomes, can be isolated. The most usual types of direct bonds are amide, disulfide, or triazole, formed as a result of using the method “click chemistry.” To regulate the optimal distance between CPP and the therapeutic agent, spacers are often used, which can be attached to CPP side chain functional groups such as the lysine amino group or the cysteine thiol group, or even the carboxyl or amino group at the C or N-terminus of the peptide, respectively [250]. There is one more strategy to construct peptide-nucleic acid complexes for gene delivery. It involves the modification of functional peptide segments on the surface of nanoparticles to create different complex nanoplatforms. Furthermore, these nanoplatforms can be used to complex nucleic acids, facilitate cellular uptake, and have multifunctional properties [251].

Cell-penetrating peptides are a well-studied group of compounds that are currently widely used as carriers for the delivery of therapeutic agents. These are short peptide sequences with a length of less than 30 amino acid residues that can pass through the cytoplasmic membrane in both volatile and non-volatile ways [252]. Even though these peptides are good at getting through cell membranes, most of these peptides cannot cross the blood-brain barrier. There are various ways in which these molecules can be classified, for example, by chemical structure, by mechanisms of entry into the cell, or by their source of origin (proteinaceous, synthetic, or chimeric) [253]. Cationic, amphiphilic, and hydrophobic peptides are distinguished by their physicochemical properties. Cationic peptides are positively charged peptides that have numerous lysine and arginine residues in their structure. While some cationic vectors can effectively bind NA at +3 charge, peptide carriers must have at least six positively charged amino acid residues [254]. The most studied CPPs are the HIV-1 TAT peptide and penetratin. These peptides act as nuclear localization sequences, which means that they can deliver various therapeutic agents to the cell nucleus by passing through nuclear pores [255,256]. Amphiphilic peptides contain both hydrophilic and hydrophobic amino acids. As a result, the charge can be positive, neutral, or negative. Hydrophobic peptides have a predominant composition of hydrophobic amino acids in their structure, such as alanine, leucine, isoleucine, phenylalanine, tryptophan, methionine, and tyrosine.

One way to directly deliver Cas9 protein and sgRNA into the cells is to use CPP. To deliver Cas9 protein, it can be conjugated to CPP via a thioester bond, while sgRNA can be complexed with CPP due to their electrostatic interactions. As a result, such delivery leads to effective gene editing and a decrease in off-target effects compared to Cas9 plasmid delivery [257]. However, the covalent bond between the anionic Cas9 protein and the cationic CPP is considered to interfere with the nuclease activity of Cas9 due to strong electrostatic interactions. Therefore, the non-covalent complexation of Cas9 with amphiphilic CPP seems to be preferable. Lostalé-Seijo and colleagues constructed an amphiphilic protein from cationic protein and hydrophobic aldehydes and showed that this structure is effective for Cas9 RNP delivery [258]. Moreover, there are other approaches to delivering Cas9-RNP into the cells using CPPs. An adaptor similar to TAT-CaM, consisting of TAT CPP and calmodulin, forms a complex with recombinant Cas9-CBS (calmodulin binding site) RNP, which is efficiently transferred into the cell [259]. In the work of Kim et al., one recombinant protein containing Cas9, NLS, and LMWP (low molecular weight protamine) was constructed. The authors reported that LMWP provides self-assembly of Cas9-LMWP/crRNA/tracrRNA complexes, as well as cell internalization in vivo [260].

The targeted peptides are commonly used to reach the desired therapeutic effect of gene therapy. The RGD (arginine-glycine-aspartic acid) motif is a specific recognition site for integrins with their ligands, which is a cell adhesion motif located on many extracellular matrix proteins (laminin, vitronectin, fibrinogen, von Willebrand factor, osteopontin, and others) and blood plasma proteins. This motif plays an important role in cell recognition and adhesion and is also used in the treatment of tumor diseases [261,262]. It is known that the αvβ3 and αvβ5 integrins are highly expressed on the surface of tumor endothelium cells compared to normal tissues [263,264,265]. Integrins are adhesion receptors, which are membrane proteins associated with extracellular matrix glycoprotein receptors on the cell surface. Integrin-ligand binding results in two types of cell adhesion: cell–cell and cell–extracellular matrix [266]. Integrins regulate such fundamental cellular processes as adhesion, migration, proliferation, and cell differentiation. Integrins also contribute to the onset and progression of many biological diseases such as angiogenesis, thrombosis, inflammation, osteoporotic neoplasia, tumor metastasis, and gene expression [267]. The distinction is made between cyclic and linear RGD peptides, while cyclic ones exhibit higher activity, as they have a less flexible conformational structure, which allows them to resist proteolysis, as well as a higher affinity for integrin receptors [268,269]. One of the best-known cyclic peptides is iRGD, which contains the RGD motif (CRGD[K/R]GP[D/E]C) and was identified through phage display screening [270]. The iRGD peptide can effectively and specifically bind not only integrins but also neuropilin-1 (NRP-1) receptors that are also overexpressed in various types of cancer [271,272]. After binding to integrin, the proteolytic cleavage of iRGD occurs, leading to the production of CRGD/K and exposing the CendR motif that interacts with NRP-1, resulting in NRP-1-dependent endocytosis [273]. This dual targeting mechanism leads to increased tumor penetration and the spread ofiRGD peptides throughout the interstitium. iRGD is currently being used as a targeting molecule of peptide-based vectors for delivery into primary leiomyoma cells [274,275].

RGD peptides are widely used in tumor therapy and have some advantages for research and practical applications. RGD is much smaller compared to monoclonal antibodies, and RGD conjugates may have easier access to tumor tissue; use of RGD minimizes the risk of immune reactivity or pathogen transmission; the synthesis of RGD peptides is a relatively simple and inexpensive process, which facilitates the transition to their use in clinical practice; RGD has a much wider range of uses than folic acid. RGDs are not only used in tumor therapy but they can also be bonded to material surfaces to control their density and orientation [276,277]. In some studies, it was demonstrated that the nanocarriers modified with the RGD peptide can effectively deliver therapeutic agents to suppress tumor growth or metastasis. Egorova and co-authors demonstrated that delivery complexes R6p-cRGD (arginine–histidine–cysteine-rich peptide R6 monomers with the inclusion of cyclic RGD-ligand) loaded with HSV-1 thymidine kinase-encoding plasmid to uterine leiomyoma cells promote decreased proliferative activity and increased the number of apoptotic and necrotic cells [278].

It is known that modification of CPPs by adding NLS peptides can be used to promote efficient nuclear entry and realize the delivery of pDNA. Yan and colleagues created a new nucleus-targeted NLS (KALA-SA) nanocarrier using monopartite NLS, a cationic CPP KALA, and stearic acid (SA). The created delivery system demonstrated enhanced cytoplasmic transport and targeted localization due to having an MP NLS. Authors have also reported that these vectors have great potential for the treatment of lung cancer [279]. It is possible to modify CPP with both NLS and RGD ligands at the same time to improve transfection efficacy. Hao and co-authors created the REDV-TAT-NLS triple tandem peptides by integrating NLS with TAT (CPPs) and REDV (RGD) to remarkably improve the targeting function for endothelial cells. They also inserted glycine sequences with various repeats into the developed complexes, and as a consequence, the functions of each peptide were synergistically performed. The produced complexes can effectively deliver the pZNF580 plasmid in endothelial cells and provide a promising option for the treatment of vascular diseases [280].

It should be noted that fusogenic peptides have a high potential for the delivery of therapeutic genes. Oliveira and colleagues investigated complexes consisting of the influenza-derived fusogenic peptide diINF-7 and siRNA targeting the epidermal growth factor receptor and the K-ras oncogenes [281]. They have shown great success in delivering these complexes into human epidermoid carcinoma cells since there was demonstrated a significant decrease in the expression of both targets. Authors have also noted that adding fusogenic peptide has a crucial role since it can help complexes successfully overcome endosomal entrapment. In recent research, it was shown that fusogenic peptides can be effectively used in gene therapy for ovarian cancer. Samec and co-authors developed and investigated three fusogenic peptide carriers, DIVA3, DIV3H, and DIV3W, composed of amphiphilic core repeats and a cationic poly(D-arginine tail, for delivering siRNA against casein kinase 2 alpha 1 (CSNK2A1) into ovarian cancer cells to reduce the aggressiveness [282]. According to the findings, using DIV3W-siCSNK2A1 complexes resulted in 94% knockdown of CSNK2A1 mRNA in vitro. Experiments in vivo exhibited that intratumoral delivery of these complexes to subcutaneous ovarian tumors not only significantly suppressed tumor growth and migration of tumor cells but also led to reducing CSNK2A1 mRNA and CK2α protein expression [282]. Cantini and colleagues demonstrated that a chimeric peptide delivery system based on the combination of a fusogenic peptide and CPPs can be potentially used to deliver siRNA selectively into cancer cells. They designed and investigated a peptide-based nanocarrier that included synthetic influenza virus-derived endosome-disruptive fusogenic peptide sequence and a stretch of cationic cell-penetrating nona-D-arginine residues [283]. The complexes were loaded with siRNA that targeted an oncoprotein CIP2A and were used to deliver it into oral cancer cells. Authors have shown that using these complexes results in increased amounts of siRNAs and significantly suppressed CIP2A protein in the cancer cells. In addition, it was not accompanied by cytotoxicity and led to a decrease in the invasiveness of cancer cells. In addition, for siRNA delivery, pH-sensitive polypeptides based on lysine, phenylalanine, and histidine were studied. It has been reported that the presence of histidine reduces the toxic properties of polyplexes [284].

Taken together, peptide-based delivery systems are commonly used nanocarriers for delivering therapeutic nucleic acids. The appropriately designed functional peptide nanoplatforms can effectively condense nucleic acids to protect them from enzyme degradation and release them at the target site. Further investigations of these nanocarriers can expand our understanding of their effective application in gene therapy for different diseases.

### 3.3. Polymeric Carriers

**Figure 2:**
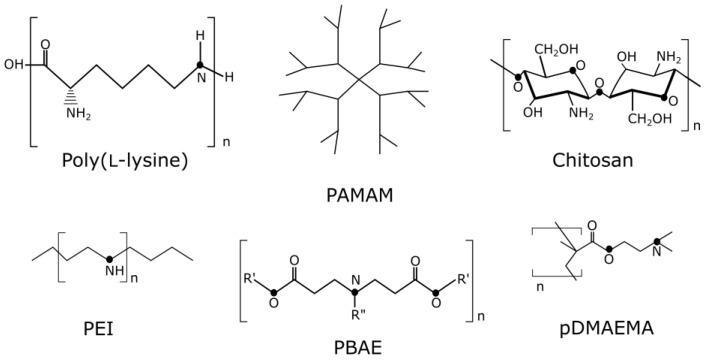
Examples of polymeric carriers for non-viral NA delivery. Dots indicate the location of signed atoms (O, N).

As it was mentioned above, polyplexes are a type of non-viral complex for gene delivery that is formed by nucleic acid and synthetic polymers, usually cationic (Figure 2). There is a wide variety of polyplexes depending on their chemical composition, architecture, and charge [285]. Polyanionic and non-charged polymers are sometimes used as protective coatings and as a component of polyplexes [115,286]. According to the principle of chemical structure, they are divided into natural NA-binding polymers (disordered and moderately branched), dendrimers (ordered and hyperbranched), and polysaccharides (such as chitosan, dextran, etc.) (reviewed in [287]).

#### 3.3.1. Polyethylenimine

Polyethylenimine (PEI) is among the most widely studied polycations used in gene therapy research [288] (Figure 2). It contains positively charged protonated amino groups that allow nucleic acids to bind. There are many forms of PEI, such as the following: different sizes (molecular weight) and branching (linear and branched). Linear PEI (LPEI) contains only primary and secondary amino groups, while branched PEI (BPEI) also contains tertiary ones. LPEI and BPEI have different syntheses. In addition, LPEI and BPEI have different physical properties, such as aggregation at room temperature and different solubilities in solvents (reviewed in [289]).

PEI is classified in accordance with its molecular weight as low molecular weight (less than 25 kDa) or high molecular weight (greater than 25 kDa). Molecular weight correlates with cytotoxicity and transfection activity. The larger the PEI molecular weight, the higher its cytotoxicity and the higher its transfection efficacy (reviewed in [290]). Cytotoxicity is caused by excessive positive surface charge, which in turn results in membrane disruptions and high affinity for negatively charged serum proteins (reviewed in [291]).

To reduce cytotoxicity and improve transfection activity PEI modifications are being developed.

Low molecular weight (LMW) PEI appears to have poor transfection efficacy due to insufficient cargo protection and weak cellular binding [292], so it must have special biodegradable bonds that could be cleaved under intracellular conditions. Zhang and colleagues [292] used acrylate-terminated orthoester rings to form HMW PEI (about 40 kDa), which readily decomposes under slightly acidic conditions. Additionally, this modified PEI is enriched with secondary amines, which improve its buffering capacity. Zhan and colleagues [290] developed glutathione-modified LMW PEI with higher in vitro transfection efficacy than “naked” PEI, especially in the presence of serum.

Another strategy for improving the transfection capacity of PEI is a modification with various bioorganic molecules, such as amino acids and fatty acids. Tyrosine-modified LMW-branched PEI has been shown to enhance siRNA delivery to tumor cells [293]. These results are of particular value because NA delivery into cells using LMW PEI is less effective than HMW PEI [294]. The mechanism of exactly how these modifications facilitate transfection activity is still unclear. The optimal balance of hydrophobicity and hydrophilicity is considered to play a key role in this phenomenon [293].

PEGylation is often used to increase biocompatibility and reduce the cytotoxic effect of PEI. Cytotoxicity can be caused by extensive polyplex aggregation as well as non-specific interactions, and PEGylation successfully solves both problems. However, the more PEGylated PEI/DNA polyplexes there are, the less effective the transfection, so an optimal variant with satisfactory cytotoxicity and significant transfection activity should be chosen. Additionally, PEG prolongs the half-life of PEI complexes in the bloodstream, reducing their affinity for serum components [295]. The combination of fatty acids and PEG-modification of LMW branched PEI allows very specific and effective delivery of genetic material to pulmonary microvasculature in vivo [296].

Occasionally, more than one type of modification is required to enhance transfection activity. Karimov and colleagues [297] used combined disulfide cross-linking and tyrosine modifications of LMW linear PEI and showed a synergetic increase in transfection efficacy compared to its unmodified or monomodified variants. Hydrophobicity is considered to promote cell uptake of PEI polyplexes and reduce cytotoxicity. Combined modification of PEI with cholesterol and perfluorinated moieties increases hydrophobicity and improves biocompatibility and transfection efficacy [298].

Alkylcarboxylation was used to reduce the cytotoxicity of HMW PEI. The anionic coating containing carboxyl groups increases the viability of transfected cells and transfection activity [299].

To achieve better transfection capacity, PEI-entrapped gold nanoparticles modified with PEG and RGD peptides were synthesized. The reduced positive charge and more appropriate 3D structure due to interior gold nanoparticles are considered to improve the transfection ability of these complexes [300].

One strategy is to shield the excess positive charge on the surface of PEI complexes. LPEI polyplexes shielded with liposomes have better transfection quality than linear PEI itself. Hiding a high charge density with a liposome provides better biocompatibility [301]. Alginates and other anionic polysaccharides are also used as coatings for PEI polyplexes. These covered complexes can be bonded with calcium ions to achieve better stability. The authors showed improved complex accumulation in tumors, stability, and the ability to transfect in vivo [291].

Researchers sometimes use two or more non-viral vectors together to consolidate their features for improved transfection efficacy. Thus, the copolymerization of PLL and PEI significantly increases the efficacy of transfection and also reduces its cytotoxicity [302]. Occasionally, complexes have a complicated composition and contain a large number of chemicals bound to each other both covalently and non-covalently. For instance, Lvand coworkers [303] synthesized nanoparticles consisting of LMW PEI grafted to epsilon-amino groups of PLL, cyclodextrin, and PEG. All these components provide good biodegradability and serum stability.

#### 3.3.2. PBAE–Poly(β-amino esters)

PBAE refers to a polymer synthesized from amines and acrylates without side products from Michael’s addition (Figure 2). A large PBAE library has been constructed for various purposes of gene delivery [304]. PBAEs are divided into two types: linear and branched [305]. Tertiary amino groups and ester bonds give PBAE good pH sensitivity and biodegradability, respectively [306]. PBAE was used by Wang and colleagues [307] as one of the nanocarrier modules along with HA and CPP for the treatment of melanoma. The resulting nanocarrier combines the penetrating epidermal penetration ability of CPP, the pH sensitivity of PBAE, and HA as a specific ligand. Guo and co-workers synthesized PBAE nanocomplexes coated with HA, which shields their positive charge. However, HA can affect the polyplex/cell interaction by hiding its positive charge. HA with grafted dopamine solves this problem due to the high bioadhesiveness of dopamine [306]. Coating of PBAE/siRNA polyplexes with guanidinylated O-carboxymethylchitosan improves their transfection activity [308].

#### 3.3.3. pDMAEMA–Poly(2-dimethylamino)ethyl Methacrylate

pDMAEMA contains only tertiary amines and, when used for nucleic acid delivery, can achieve a transfection efficacy of 90% compared to PEI [309] (Figure 2). There are two issues affecting pDMAEMA transfection efficacy: tight nucleic acid binding (poor cargo release) and stability under physiological conditions. In their work, Cheng et al. solved these problems by synthesizing pH-sensitive nanocarriers. Under physiological conditions, the complexes are hydrophobic due to the specific interaction of p(OEGMA-DMAEMA) with p(PMA-PMBA), which allows these complexes to be stable in the presence of serum. However, cleavage of benzoic imines at low pH (in endosomes) reduces the hydrophobicity of the complexes, allowing for easy nucleic acid release [310]. pDMAEMA can be conjugated with dextran to provide a biodegradable and non-toxic carrier. However, such complexes are not serum-resistant, so it is essential to improve this characteristic. Molecules with a large number of hydroxyl groups avoid interactions with serum proteins. One example of such molecules is the polymer of lactobionamidoethyl methacrylate [311]. There is also a work in which magnetic iron oxide nanocubes are coated with pDMAEMA [312].

#### 3.3.4. Dendrimers

Dendrimers are highly branched polymers with a tree-like ordered structure for drug delivery (including gene delivery) synthesized by repeating the assembly of several layers around the core molecule through covalent conjugation [313].

- PAMAM–Polyamidoamine

The most common dendrimer used for nucleic acid delivery is PAMAM (PolyAMidoAMine) (Figure 2). PAMAM generation refers to the number of sequential steps by which PAMAM was synthesized. The more PAMAM is generated, the greater its molecular weight, surface charge, and, as a result, cytotoxicity [314]. On the one hand, PAMAM has a higher transfection efficacy, better flexibility, and lower immunogenicity than other polymeric carriers; on the other hand, it has significant cytotoxicity and rapid clearance in the bloodstream [309]. Cytotoxicity is caused by the extremely positive charge on the surface of PAMAM due to the terminal amino groups [315]. To eliminate these shortcomings, modifications are being developed that are usually grafted onto terminal primary amino groups due to their high reactivity [316] (Table 1).

Pishavar and colleagues used three modifications—alkylcarboxylation, PEGylation, and the addition of cholesteryl chloroformate—in different ratios. These modifications are considered to improve the hydrophobicity/lipophilicity balance of complexes [315]. Another way to improve transfection efficacy is to make carriers specifically sensitive to the environment. For instance, ROS-responsive compounds are one such solution, as they reduce the surface charge of the complexes and promote the nucleic acid release. Mainly, ROS-responsive carriers are used for tumor delivery due to the high concentration of ROS inside tumor cells. As an example, thioketal is known to be cleaved under ROS conditions and is used as the core of ROS-sensitive PAMAM [324].

To reduce the clearance of dendriplexes in blood circulation, along with PEGylation, zwitterionic modifications such as carboxybetaine and acrylamide are also used [319,320].

To increase the transfection activity of low-generation PAMAM, imidazolyl and guanidyl are used. The combination of these two chemical groups in 2-aminoimidazole can promote PAMAM transfection capacity by increasing the“proton sponge effect”, improving DNA condensation, and improving cell binding due to the formation of hydrogen bonds between the phosphate groups of DNA and the outer surface of the cell membrane [324].

Dendriplexes can be non-covalently encapsulated in anionic liposomes to effectively shield excess PAMAM primary amines (lipodendriplexes). Lipodendriplexes exhibit better stability in the extracellular space and better bind to cells than “naked”dendriplexes [321]. Hybrid lipodendriplexes can also be assembled from PAMAM coated with phospholipids and surfactant molecules. Phospholipids facilitate cell binding due to their greater lipophilicity and increase the stability of polyplexes [325]. Proper 3D conformation of dendriplexes is also essential for significant transfection activity. The ball-shaped form is believed to allow greater DNA condensation and, as a consequence, better compensation of terminal amino groups. To properly maintain the 3D conformation, gold nanoparticles can be used [320]. Uncharged liposomes can be decorated with positively charged G0 PAMAM to obtain cationic liposomes that are well suited for membrane fusion, DNA condensation, and cell binding through the protonated positively charged amino groups of PAMAM [322].

There is a new technology for assembling supramolecular PAMAM called host-guest assembly. It is based on the non-covalent interaction of covalently bound host (β-cyclodextrin) and guest (adamantine) molecules to PAMAM. This method makes it easy to produce high molecular weight monodisperse carriers with better transfection activity [326].

PAMAM polyplexes can be targeted to certain tissues for more specific gene therapy. For instance, skeletal muscles can be targeted by the special targeting peptide ASSLNIA [98]. Hydroxylation of PAMAMs allows them to pass through the blood-brain barrier. PAMAM modifications with monosaccharides such as glucose and mannose improve their ability to enter glioblastoma cells due to overexpression of the corresponding sugar transporter in that tumor [323].

In addition, PAMAM can be used for Cas9-RNP delivery. PAMAM modified with phenylboronic acid was used for successful intracellular delivery of the Cas9 direct editing system using dendrimers. Phenylboronic acid can bind to various chemical groups over the entire surface of the protein and thus facilitate the assembly of nanoparticles. Consequently, this modified PAMAM can also be used to deliver other proteins with different pI and molecular weights [327].

- Other types of dendrimers for gene delivery

1. Cationic phosphorus dendrimers (CPD). This type of dendrimers has a backbone consisting of aminothiophosphates at each branch point. Additionally, cationic phosphorus dendrimers possess a great potential to be altered (varying hydrophobicity/hydrophilicity) for certain goals. However, nucleic acid binding by unmodified CPD is poor. CPD grafted with pyrrolidinium or morpholinium containing secondary amines exhibits significant nucleic acid binding [328]. Ihnatsyeu-Kachan and co-workers utilized piperidine-terminated CPD for pro-apoptotic siRNA cocktail delivery into the HeLa cell line [329].

2. Carbosilane dendrimers (CD). CDs contain silicon atoms in the molecular skeleton. Many functional groups can be added to carbosilane for various purposes using silicon chemistry. Unmodified carbosilanes alone cannot provide significant transfection due to the absence of a positively charged group on their surface [330]. Positively charged groups such as ammonium and guanidine [331] can be attached to the CD surface for successful nucleic acid condensation. Herma and colleagues synthesized trimethylphosphonium-terminated CDs for siRNA delivery and showed similar transfection efficacy and much lower cytotoxicity compared with the trimethylammonium-terminated analog [332]. CDs can be used for coating silver and gold nanoparticles for siRNA delivery in vitro [333,334].

3. PPI—Polypropyleneimine. PPI dendrimer is smaller, stiffer, and more compact than PAMAM dendrimer of the same generation. There are many modifications used to increase the transfection activity of PPIs and reduce cytotoxicity. Alkylcarboxylated PPIs can be successfully used for the co-delivery of genes and drugs (e.g., doxorubicin and the TRAIL plasmid) in tumors [335]. Based on polypropyleneimine, another co-delivery system for cancer has been created. For antitumor therapy, cholesterol-bearing PEGylated PPI-based self-assembling vesicles (dendrimersomes) with reducible disulfide bonds were obtained [336].

4. Poly(L-lysine) dendrimers (PLDs). PLDs are PLL-based dendrimers that are more biodegradable and water-soluble than PAMAM [337]. However, PLDs are extremely toxic due to the abundance of cationic groups.To decrease their cytotoxicity, anionic polymers such as γ-polyglutamic acid can be used [338]. Gorzkiewicz and co-workers synthesized lysine-based peptide dendrimers for cell transfection. When two lysine-based peptide dendrimers are compared, the dendrimer with lysine monomers between branching points is more effective but still more cytotoxic than the dendrimer with glycine at the same point [339].

#### 3.3.5. Carbohydrates

Chitosan is a linear polysaccharide composed of *N*-acetylated glucose and beta (1–4) linked repeats of 2-amino-2-deoxy-D-glucose (Figure 2). Chitosan, being a biological molecule, demonstrates high biodegradability and non-toxicity, as well as significant NA condensation because of its amino groups, while its solubility at physiological pH values is low [340]. Chitosan is obtained from chitin as a result of its deacetylation [309].

It is reported that *O*-carboxymethylation and guanidinylation of chitosan improve the biological and physiochemical properties of the complexes with this polymer [308]. Unmodified chitosan has a low ability to escape from the endosome due to low proton buffering capacity. This carrier can be combined with other polymers to achieve high biocompatibility while also allowing for significant endosomal escape [341]. To enhance the colloidal stability and solubility of chitosan/siRNA complexes, negatively charged carboxymethyldextran can be used [341]. For the water solubility of chitosan, it was modified with thiolated trimethyl, 4-*N,N*dimethylaminobenzyl, or *O*-carboxymethyl groups [342].

### 3.4. DNA Nanostructures as Carrier Systems

The utilization of DNA nanostructures is nowadays considered a promising approach for delivering therapeutic cargo. These delivery systems can be modified and used in different directions, such as therapy for some diseases. DNA nanostructures are mainly used to implement gene therapy approaches based on RNA delivery.

One of the most common methods for creating molecular nanostructures of any shape is the DNA origami method, which was introduced in 2006 by P.W. Rothemund [343]. This method is based on the process of interaction between a long “scaffold” single-stranded DNA and hundreds of short “staple” ssDNA that are complementary to different parts of the long strand. At the first stage of the assembly, it is determined where the short chains should be located. Next, oligonucleotides are synthesized that are complementary to the determined regions. Then the long and short strands of DNA are mixed, and the mixture is heated to a certain temperature. In this way, randomly formed double-stranded regions are untwisted. During the cooling process, the oligonucleotides are connected to certain parts of the long strands; as a result, the long strands are folded into certain complex nanostructures of various shapes, such as rectangles, stars, and other figures. In this way, 2D structures are formed, and the diameter of these figures is about 100 nm. Shortly thereafter, the DNA origami method was extended into three dimensions. In 2009, Andersen and colleagues designed the famous addressable DNA box in size 42 nm × 36 nm × 36 nm with a lid that can be opened under certain conditions, specifically in the presence of externally supplied DNA “keys” [344]. To create the box, they divided the scaffold strand into several parts to form separate flat substructures and then connected these flat parts. It should also be mentioned that some special computer-aided software programs, such as “caDNAno,” “vHelix,” and GIDEON, could be used to design many different DNA nanostructures [345,346,347]. For example, they enable defining the sequences that must be synthesized to assemble two- or three-dimensional nanostructures designed by the DNA origami.

It should be mentioned that there is a scaffold-free approach to constructing complex wireframe nanostructures. DNA origami technology involves the scaffold strand as an important part, but this method is a programmable self-assembly of many small tiles or bricks into a complex designed nanostructure [348,349]. It was demonstrated that self-assembled DNA tetrahedral nanoparticles with a well-defined size can deliver siRNAs into cells and silence target genes in tumors [350]. Researchers used peptides and folate as cancer-targeting ligands and showed that gene silencing occurs only when the ligands are assembled with proper spatial localization. Furthermore, in vivo tests showed that created nanoparticles exposed the blood to longer circulation times than the parent siRNA, mostly in tumors and kidneys [350]. Wang et al. produced fully addressable wireframe structures such as tubes and polyhedrons, and also they demonstrated that using the triangulation method, these platforms become significantly rigid and not prone to deformation [351].

Bujold et al. designed, synthesized, and optimized the first functional DNA “nanosuitcase” capable of encapsulating siRNA [352]. These DNA-minimal cages are programmable and can assemble as discrete objects, according to reports. Moreover, this DNA nanostructure can effectively protect its cargo from targeted degradation and selectively release it when mRNA or microRNA oligonucleotide triggers are recognized. After recognition, the two gating strands unwind via strand displacement, releasing the siRNA cargo. Since gate strands can be involved in gate opening and gene silencing as a result of DNA nanosuitcases modification, synergistic therapy is possible [352].

Altogether, these studies showed that DNA-based nanostructures are promising nanomaterials that have shown significant potential in biomedical and biomolecular applications because of their impressive properties such as biocompatibility, programmability, and optimal stability [353,354,355]. These nanostructures can be used as biosensors or nanocarriers for cancer therapy, photodynamic therapy, and gene therapy of hereditary diseases and are also used for bioimaging and biodetection [356,357,358,359].

### 3.5. Other Types of Carriers

#### 3.5.1. Microvesicles and Exosomes

There are naturally occurring vesicles called exosomes and microvesicles that are produced by certain types of cells. These vesicles have been demonstrated to contain genetic information such as mRNA and non-coding RNA in vivo. The distinctions between exosomes and microvesicles are based on biogenesis and several properties. Exosomes result from the release of intralumenal vesicles of multivesicular bodies into the environment, while microvesicles are gained from the direct budding of the plasma membrane. In addition, exosomes are less heterogeneous, and their diameter varies from 40 to 100 nm, while the diameter of microvesicles varies from 50 to 1000 nm. Due to the ability of these structures to transfer nucleic acids, they can be used as carriers in gene therapy [360].

#### 3.5.2. Exosomes in Gene Therapy

Tumor-produced exosomes can deliver CRISPR/Cas9 plasmid to the tumor tissue in vivo. Exosomes obtained from tumor cell cultures were electroporated with the plasmid against the PARP1 gene and injected intravenously into xenograft mice. After the delivery of exosomes, efficient gene editing has been shown along with less immunogenicity and greater specificity for tumor tissue [361]. Alvarez-Erviti and colleagues used exosomes produced by bone marrow cells to deliver siRNA against BACE1 to the brain and muscles in vivo. Murine bone marrow was harvested and transfected with a plasmid encoding chimeric proteins. The protein consists of Lamp2b (an exosomal transmembrane protein) and one of the ligands for targeted delivery to the brain and muscle. Purified exosomes were then subjected to electroporation with the anti-BACE1 siRNA. The authors show no knockdown effects in off-target tissues such as the liver and kidneys. Thus, exosomes are programmable carriers for specific delivery to certain tissues [362].

As mentioned earlier, exosomes range in size from 40 to 100 nm. Therefore, they can be loaded with small cargo such as siRNAs, but they have a problem loading fairly large plasmids such as the Cas9-expressing plasmid. This problem was solved by incubating exosomes and liposomes. Lin Y. et al. created an exosome-liposome hybrid by fusion for CRISPR/Cas9 plasmid delivery. Liposome–exosome hybrids were generated by incubating plasmid-loaded exosomes and liposomes and, despite being larger than both liposomes and exosomes, had a significant synergetic effect [363]. However, there are works reporting successful loading of the CRISPR/Cas plasmid into exosomes despite limitations [361,364].

#### 3.5.3. Microvesicles in Gene Therapy

He C. et al. used epithelial-derived microvesicles to treat cancer. The researchers created cell cultures that stably express Cas9 protein and harvested their microvesicles. Microvesicles electroporated with the sgRNA plasmid have been shown to have an anti-cancer effect. The authors compared the efficiency with which cancer cell-derived and epithelial-derived microvesicles delivered the CRISPR/Cas9 system into cancer cells. Surprisingly, cancer cell-derived microvesicles were less effective than microvesicles derived from the epithelium. Probably, microvesicles originating from cancer cells can promote cancer proliferation in target cells [365].

There are several types of extracellular vesicles (EVs) that can be used for Cas9-sgRNA delivery. Exosomes are EVs with a diameter of 50–150 nm that are produced by the release of intraluminal vesicles with multivesicular bodies in almost all types of cells under specific conditions. There are many ways to produce these carriers [366]. Special “cell factories” are used to produce exosomes containing a variety of medicines, including Cas9/sgRNA ribonucleoproteins [121]. Ye and colleagues used HEK293T cell lines, which contained exosomal membrane protein CD63 conjugated with GFP. The GFP antibody linked to Cas9 protein can bind to CD63-GFP; therefore, selective packaging of Cas9 protein in exosomes is achieved [366].

Gesicles are EVs produced by budding from the plasma membrane in cells overexpressing vesicular stomatitis virus G glycoprotein. Campbell and co-workers used HEK293FT cells to produce gesicles containing the Cas9 ribonucleoprotein. The authors showed a successful Cas9-RNP package and a significant effect of delivery of the editing system in CHME-5 cells, leading to the cleavage of HIS provirus [367].

### 3.6. Nanomaterials as Non-Viral Carriers

#### 3.6.1. Graphene-Based Nanomaterials

Graphene-oxide (GO) is a product of graphite treatment with a strong oxidizer and acids. It is one atom thick (about 1.1 nm) and has several functional groups such as epoxy, carbonyl, hydroxyl, and phenol groups [368]. Functional groups facilitate the binding of some drugs and single-stranded DNA, while double-stranded DNA binds to GO poorly. In addition, GO has negative charges that could interfere with the interaction between GO and DNA [369]. One strategy for improving GO capacity is a modification with other non-viral vectors. Functional groups are good targets for modification because of their high reactivity [368]. For instance, Kim and colleagues added LMW BPEI to carboxyl groups of GO and achieved high transfection efficacy with relatively low cytotoxicity [369].

Kim H. and Kim W. J. demonstrated the photothermal transfection ability of reduced GO. Reduced PEI-modified GO possesses high endosomal escape ability due to (1) the proton sponge effect of PEI and (2) the photothermal effect of reduced GO. Reduced GO in the near infrared region of light accumulates heat, resulting in endosomal membrane destruction and the release of nanocomplexes [370]. The conjugation of PEI and PEG with GO through disulfide bonds increases DNA dissociation in the cytoplasm and, consequently, the efficacy of transfection [371].

#### 3.6.2. Nanotubes

Carbon nanotubes (CNTs) are cylindrical graphene sheets with unique physicochemical properties that allow them to effectively cross the plasma membrane. There are both single-walled nanotubes (SWNTs) and those consisting of several coaxial walls (multi-walled nanotubes, MWNTs) [372]. For efficient DNA binding and transfection, CNTs should be coated with polyamines such as PEI or PAMAM. Interestingly, CNTs lack the functional groups for adding modification, but polyamines are adsorbed on CNTs due to hydrophobic interactions [372], or CNTs could be carboxylated and then conjugated with polyamines [373]. Nia and colleagues [374] synthesized PEI conjugated with carbon nanotubes. This modified nanocarrier combines the advantages of PEI (great endosomal escape) and CNT (good cell entering) and has a much higher transfection efficiency. Modifications prevent CNT aggregation and promote DNA condensation through electrostatic interaction. Both SWNTs [375] and MWNTs [373] demonstrate the ability to efficiently transfect. Modified CNTs are capable of delivering oligonucleotides [372] as well as plasmid DNA [376]. CNTs can be functionalized with pyridine, which enhances nucleic acid binding via hydrogen bonds and stacking interactions. The grafting of CNTs with Fe_3_O_4_ magnetic groups for magnetofection was demonstrated [377].

#### 3.6.3. Mesoporous Silica Nanoparticles

Mesoporous silica nanoparticles (MSNs) have a strong framework with a porous structure and a large surface area, which allows the attachment of various functional groups. Chemically, MSNs have a honeycomb-like structure and an active surface. Chemically active surfaces are easily functionalized, allowing MSNs to package nucleic acid [378].

Unmodified MSNs have a negative net charge due to silanol groups and must be supplemented with positively charged groups [378]. Zarei and colleagues improved MSN transfection efficiency by coating them with a PEI. MSNs were phosphonated and then covered with PEI. Electrostatic interaction with phosphonate groups on the surface of MSNs results in PEI coating [379]. Slita and colleagues covered pore walls with hyperbranched PEI for amino group enrichment. After complexing with siRNA, one more PEI layer was added for nucleic acid protection and cellular uptake improvement [380]. MSNs can be grafted with several modifications, such as cationic polymers, to improve condensability and biocompatibility. In the work of Nhavene and colleagues, biodegradable polymers such as chitosan and polycaprolactone with MSNs were linked through the silanol group [381].

Wang Y. et al. synthesized sophisticated MSN-based nanoparticles for the treatment of tumors in vivo. Amino-functionalized MSNs adsorbed siRNA and miRNA and were then loaded with indocyanine green as a photosensitizer for endosomolytic activity. The loaded MSNs were stabilized with an iRGD ligand-modified lipid layer. Lipid molecules were conjugated to iRGD via copper-free click chemistry. The nanoparticles possess the ability for efficient tumor penetration as well as an endosomal escape [382].

To summarize, there are several challenges to successful non-viral nucleic acid delivery, and a variety of modifications have been proposed to overcome them. There are the main problems and their possible solutions:

1. Reducing cytotoxicity and enhancing biocompatibility. (a) Shielding of positive surface charge with an anionic molecule. (b) Using natural biological molecules such as dextran and hyaluronic acid.

2. Addressing the problem of non-specific elimination of polyplexes by the liver and early elimination. It can be done using systems that target a selected organ, bypassing the liver [383,384].

3. Enhancement of cargo (nucleic acid) release. (a) Creation of cleavable carriers under intracellular conditions (disulfide bonds, glutathione bonds) for efficient intracellular delivery of pDNA [385] and mRNA [386].

4. Improvement of endosomal escape. (a) Compound complexes are assembled using high proton-buffering capacity carriers such as PEI [72] or additional glutamic acid or histidine moieties [387].

## 4. Conclusions

Currently, non-viral NA delivery research is an actively developing area. We are at a turning point in the development of science when the main barriers to complexes have already been well studied, the main targeting molecules for different tissues have been found, and the strategies of molecular genetic influence allow choosing the most effective method of gene therapy. Due to their high efficiency, viral vectors have been the only way to deliver therapeutic constructs for a long time. However, some non-viral carriers are now showing impressive transfection results, which makes the competition between viral and non-viral approaches to gene delivery even stronger. Further development in this direction clearly tends to expand the accumulated knowledge and search for more specific ligands and modifications that improve the properties of the carriers and their transfection ability.

## Figures and Tables

**Table 1:** PAMAM modifications.

Problem Being Solved	Modification	References
Hydrophobicity/lipophilicity balance	Alkylcarboxyl, PEG, cholesteryl chloroformate	[315]
Enhancement of biodegradability	GLFG oligopeptide	[317]
	Thioketal core	[318]
Half-life blood circulation prolongation and clearance reducing	PEG attached through disulfide bond	[319]
	Carboxybetaine acrylamide	[320]
	Lipodendriplexes	[321]
Improvement of cell binding and nucleic acid binding	Gold nanoparticle core	[320]
	PAMAM-coated liposomes	[322]
Tissue specificity	ASSLNIA oligopeptide	[98]
	Monosaccharides (glucose, mannose)	[323]

## Data Availability

Not applicable.
